# Supplementary figures and images for: Cerebral compliance assessment from intracranial pressure waveform analysis: Is a positional shift-related increase in intracranial pressure predictable?
Source: PLoS One. 2024 Dec 30;19(12):e0316167. doi: 10.1371/journal.pone.0316167 (PMC11684684; doi:10.1371/journal.pone.0316167)

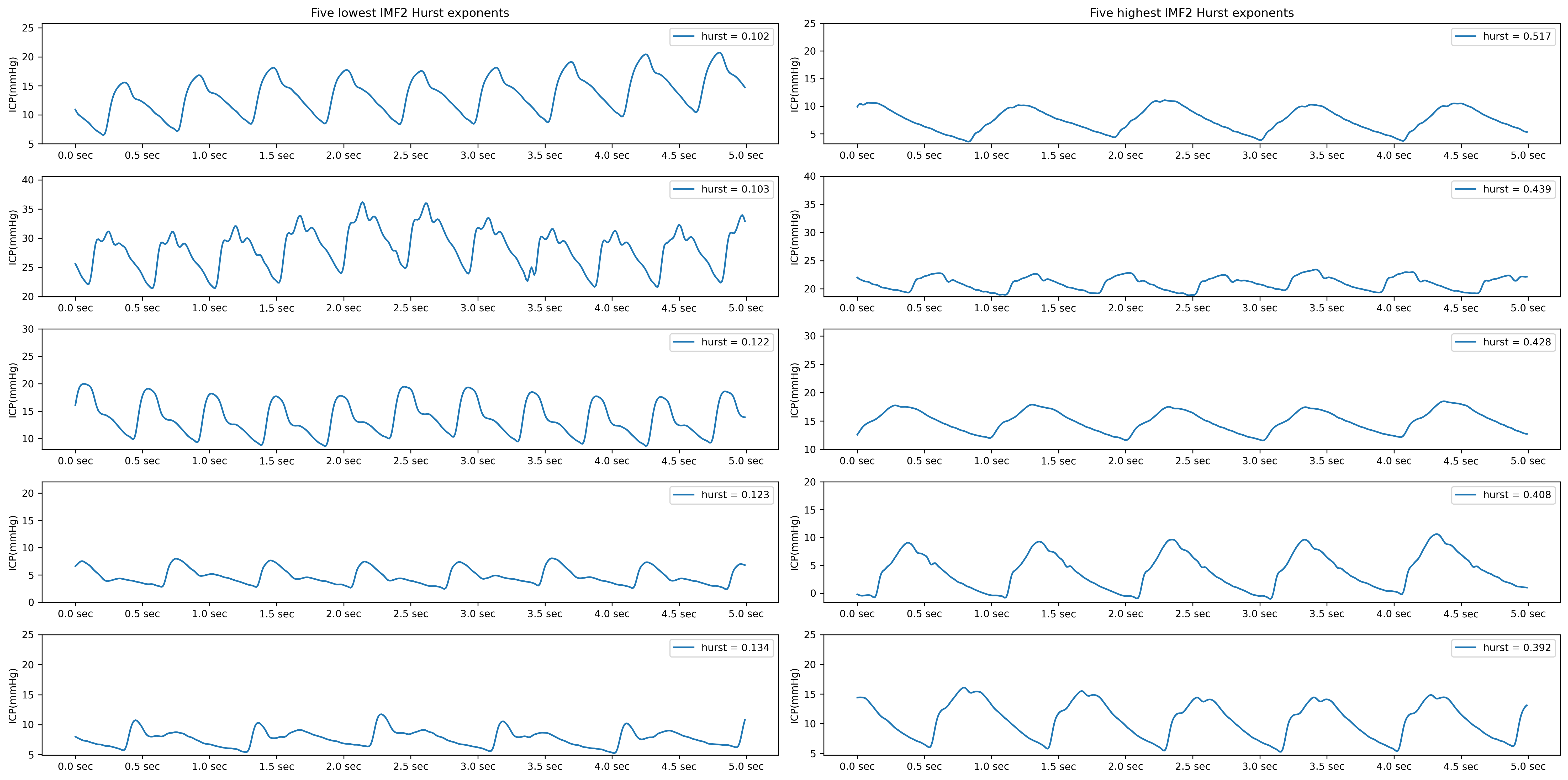

Supplement: S1 Fig — Left column: Five lowest Hurst exponents over the whole dataset. Right column: Five highest Hurst exponents over the whole dataset. (TIFF) [file pone.0316167.s001.tiff]

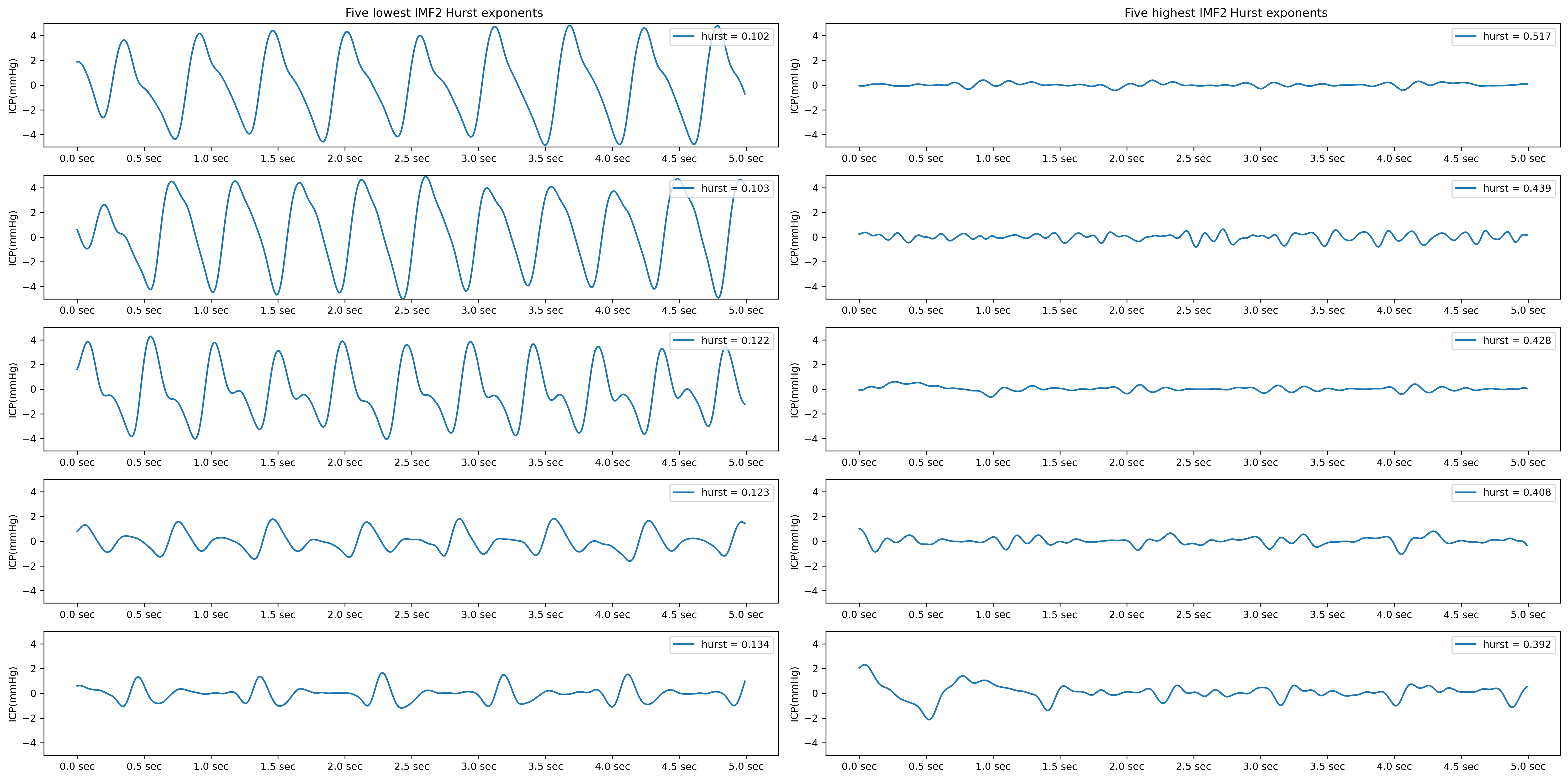

Supplement: S2 Fig — Left column: Five lowest Hurst exponents over the whole dataset. Right column: Five highest Hurst exponents over the whole dataset. (TIFF) [file pone.0316167.s002.tiff]

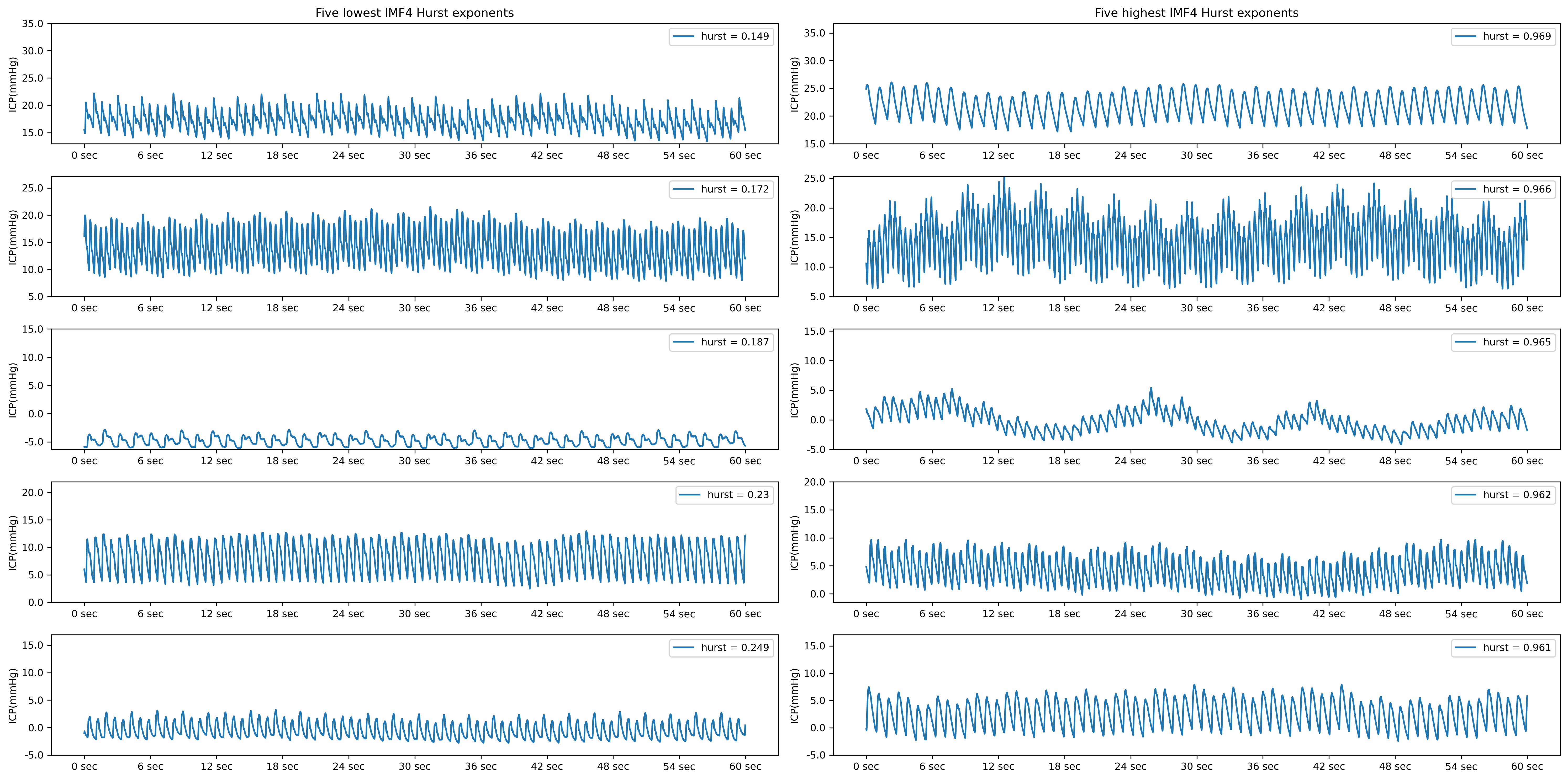

Supplement: S3 Fig — Left column: Five lowest Hurst exponents over the whole dataset. Right column: Five highest Hurst exponents over the whole dataset. (TIFF) [file pone.0316167.s003.tiff]

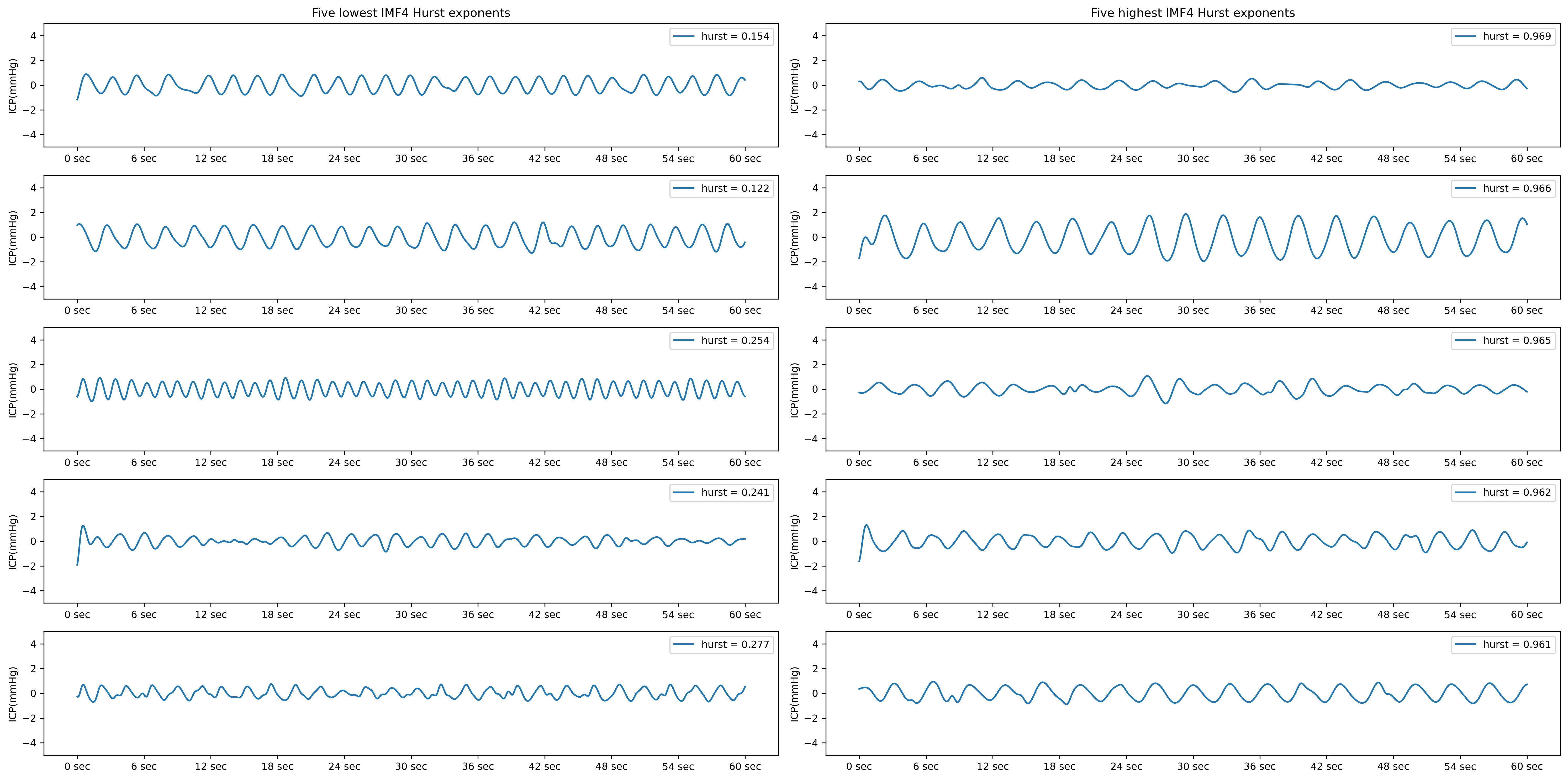

Supplement: S4 Fig — Left column: Five lowest Hurst exponents over the whole dataset. Right column: Five highest Hurst exponents over the whole dataset. (TIFF) [file pone.0316167.s004.tiff]

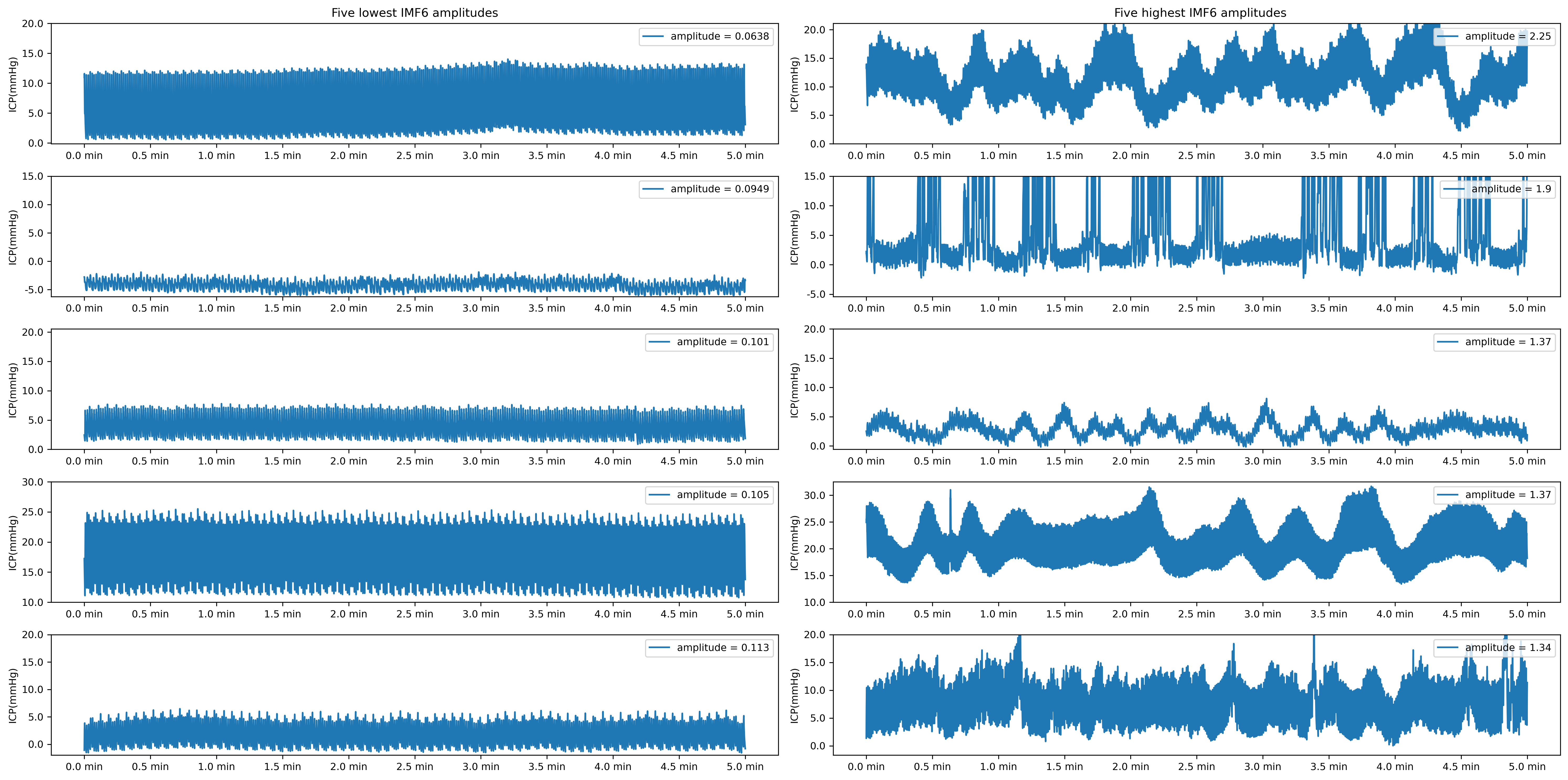

Supplement: S5 Fig — Left column: Five lowest amplitudes over the whole dataset. Right column: Five highest amplitudes over the whole dataset. (TIFF) [file pone.0316167.s005.tiff]

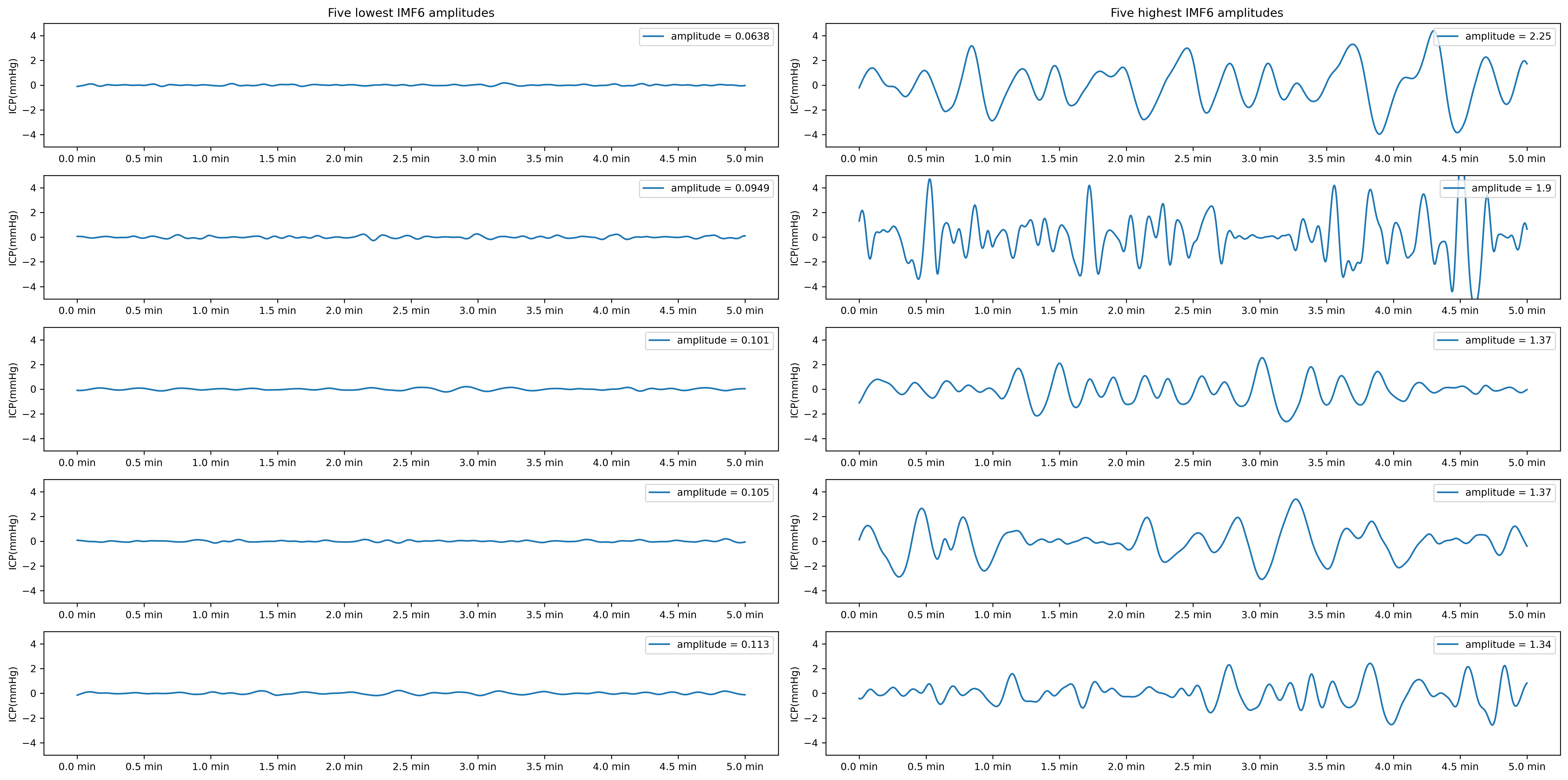

Supplement: S6 Fig — Left column: Five lowest amplitudes over the whole dataset. Right column: Five highest amplitudes over the whole dataset. (TIFF) [file pone.0316167.s006.tiff]
