# Supplementary material for: Cerebral compliance assessment from intracranial pressure waveform analysis: Is a positional shift-related increase in intracranial pressure predictable?
Source: PLoS One. 2024 Dec 30;19(12):e0316167. doi: 10.1371/journal.pone.0316167 (PMC11684684; doi:10.1371/journal.pone.0316167)
Supplement: Attachment — Submitted filename: Response to Reviewers.docx [file pone.0316167.s008.docx]

**Response to Reviewers**

Dear Editor,

Thank you for the opportunity to review our manuscript "Cerebral Compliance Assessment from Intracranial Pressure Waveform Analysis: Is a Positional Shift-Related Increase in ICP Predictable?" submitted for publication in PlosOne. We sincerely appreciate the very positive and constructive feedback from the reviewers. In addition to the minor linguistic adjustments mentioned in the Editor's comments, please find below the few changes that have been made to the manuscript to adress the suggestions made by the reviewers.

---

**Please, include information on patients' outcomes into a revised manuscript.**

We included in the cohort description (Table 1) the 28-day mortality as well as the length of stay in an Intensive Care Unit.

**Your results show that just a few patients had a low compliance. All others were in flat zone of intracranial pressure -volume curve. Please, include comment on that in order not to create a wrong impression of paper's readers that P2/P1 ratio is not associated with intracraniospinal compliance changes.**

We integrated a paragraph in the discussion to precise our findings concerning the P2/P1 ratio (lines 413 – 419). As explicitely stated, our results do not call into question the relevance of the P2/P1 ratio:

*It is noteworthy that the P2/P1 ratio, classically used to characterise the ICC via the pulse waveform, was not selected in the final classification model. Its absence should not be interpreted as a challenge to its well-established clinical relevance. In fact, this index had a high Spearman correlation with the two selected IMF2-related features, i.e., 0.71 for the Hurst exponent and -0.37 for the frequency centroid. These results suggest that the information provided by the P2/P1 ratio was already embedded in these two ICP signal features.*

---

We hope that the revised manuscript addresses all the reviewers' concerns, and that is it now suitable for publication in PlosOne.

Thank you once again for your consideration. We look forward to your positive response.

Sincerely,

Donatien Legé

Pierre-Henri Murgat

Russell Chabanne

Kevin Lagarde

Clément Magand

Jean-François Payen

Marion Prud'homme

Yoann Launey

Laurent Gergelé
